# Initial therapeutic target attainment of perampanel in pediatric patients with epilepsy

**DOI:** 10.3389/fphar.2023.1209815

**Published:** 2023-11-15

**Authors:** Lingyan Yu, Meng Chen, Jieqiong Liu, Zhenwei Yu, Jianhua Feng, Haibin Dai

**Affiliations:** ^1^ Department of Pharmacy, Second Affiliated Hospital, School of Medicine, Zhejiang University, Hangzhou, Zhejiang, China; ^2^ Department of Pharmacy, 903 Hospital of the Joint Logistic Support Force of the PLA, Hangzhou, Zhejiang, China; ^3^ Department of Pharmacy, Sir Run Run Shaw Hospital, College of Medicine, Zhejiang University, Hangzhou, Zhejiang, China; ^4^ Department of Pediatrics, Second Affiliated Hospital, School of Medicine, Zhejiang University, Hangzhou, Zhejiang, China

**Keywords:** epilepsy, seizure, pediatric, perampanel, therapeutic drug monitoring

## Abstract

Perampanel is a promising option for the treatment of pediatric epilepsy, but its plasma concentration varies among patients. This retrospective study aimed to investigate the initial target attainment of perampanel plasma concentration in pediatric patients with epilepsy in China. Inpatients admitted from January 2020 to December 2021 in a tertiary hospital were retrospectively included according to pre-set criteria. Demographic characteristics of patients and dosing strategies and therapeutic drug monitoring results were collected. A total of 137 pediatric patients (84 females and 53 males, aged from 0.6 to 16.4 years) were include for analysis. The perampanel concentrations varied greatly from 60 to 1,560 mg/L among patients, but 89.8% had suitable perampanel concentrations (100–1,000 ng/mL). The concomitant use of enzyme-inductive antiepileptic drugs (AEDs) was the only identified risk factor associated with target nonattainment (OR = 5.92, 95% confidence interval 1.68–20.9). Initial perampanel target attainment in pediatric patients is satisfactory. Routine therapeutic drug monitoring to achieved the suggested concentration range for these patients may be unnecessary, except for those receiving combined enzyme inductive AEDs.

## Introduction

Epilepsy is a common condition affecting children with a high prevalence. It has been reported that 1 out of 150 children will have a diagnosis of epilepsy in the first 10 years of life (Aaberg et al., 2017). More seriously, approximately 25% of patients with epilepsy are drug resistant (Sultana et al., 2021). Many different comorbidities may affect these patients. Cognitive and neuropsychiatric disorders such as attention deficit with hyperactivity disorder, autism spectrum disorders, and neurobehavioral problems in children are more common than in the general population (Coppola et al., 2019). The heterogeneity of seizures and epilepsies, the coexistence of comorbidities, and the broad spectrum of efficacy, safety, and tolerability related to the antiepileptic drugs (AEDs), make the management of these patients actually challenging (Fattorusso et al., 2021). Perampanel (PER) is a selective, noncompetitive antagonist of the ionotropic α-amino-3-hydroxy-5-methyl-4-isoxazolepropionic acid glutamate (AMPA) receptor on postsynaptic neurons. It has broad anti-seizure effects and has shown good clinical efficacy in adolescents and children, including those with drug-resistant epilepsy (Chang et al., 2020; Gao et al., 2022). Oral PER is rapidly and almost completely absorbed, with low systemic clearance and high relative bioavailability in humans (Rogawski and Hanada, 2013). PER is approximately 95%–97% bound to plasma proteins in a wide concentration range, and only 5% free PER exerted pharmacologic effect. The distribution volume of PER is large (approximately 1.1 L/kg) and the half-life is also long (105 h). PER is extensively metabolized via primary oxidation, which is mediated by CYP3A4 and/or CYP3A5, and sequential glucuronidation (de Biase et al., 2019). However, potential drug interactions, as well as other individual factors, may contribute to large fluctuations in plasma drug concentrations and, therefore, clinical response. Therapeutic drug monitoring (TDM) is an essential tool to address this complexity, enabling the definition of individual therapeutic concentrations and adaptive control of dosing to minimize drug interactions and prevent loss of efficacy or toxicity (Krasowski, 2010). Some studies have shown that PER plasma levels were affected by otherAEDs in routine TDM practice, including carbamazepine, phenobarbital, valproate and topiramate (Patsalos et al., 2016; Contin et al., 2018; Silva et al., 2023). A recent study indicated that enzyme-inductive AED could increase PER clearance (Fujita et al., 2022). However, it is still unknown whether these drug interactions have impact on PER therapeutic target nonattainment. Thus, we carried out this study to evaluate the initial therapeutic target attainment of PER and identify any independent risk factors associated with target nonattainment in Chinese pediatric patients with epilepsy.

## Methods

### Ethics and informed consent

This study was approved by the Ethics Committee of the Second Affiliated Hospital, School of Medicine, Zhejiang University (reference number 2021-YAN0406). Informed consent was waived due to the retrospective nature of the study and was in accordance with regional regulation requirements.

### Patients and data collection

Inpatients admitted from January 2020 to December 2021 in our hospital were retrospectively included according to the following criteria: (a) patients aged less than 18 years; (b) patients treated with PER as a monotherapy or adjunctive therapy for epilepsy; and (c) patients who underwent PER TDM during the study period. However, patients with poor adherence to PER were excluded. Poor adherence was defined as prescription-based proportion of days covered lower than 0.8 in the first 3 months (Osterberg and Blaschke, 2005; Chen et al., 2023). The patients’ medical records, dosage of PER, age, sex, body weight, and comedications (other AEDs). The dosing strategy was set by the physician according to its label, which was detailly described in our previous publication (Miao et al., 2023). In our hospital, PER TDM was performed 3 weeks after initiation of PER treatment, as PER would achieve steady states after 19 days of dosing. Blood samples were collected in the morning, approximately 12 h after the previous dose. The plasma PER concentration was determined by a validated HPLC method (Franco et al., 2016). Only the first TDM result was included for analysis. A range of 100–1,000 mg/L was considered the target therapeutic range in our center.

### Data analysis

The continuous results are presented as the mean and standard deviation, and the categorical results are presented as numbers and percentages. The concentration-to-dose ratio (C/D) of PER was calculated as follows: C/D (kg/L) = [PER concentration (mg/L)/PER dose per weight (mg/kg)]. To evaluate the effect of concomitant AEDs and other factors on the plasma concentration of PER, a simple univariate linear regression was performed using C/D as the dependent variable. Then, factors with *p* < 0.2 were put into a multivariate linear regression model to seek influencing factors of C/D. The included patients were subsequently divided into groups by factors. Differences in the C/D between groups were tested using Student’s t-test. To analyze independent variables associated with PER target nonattainment, a univariate logistic analysis was performed, and any factors with *p* < 0.2 were put into a backward multivariate logistic analysis. All statistical analyses were run in SPSS. Statistical significance was set at *p* < 0.05.

## Results

**FIGURE 1:**
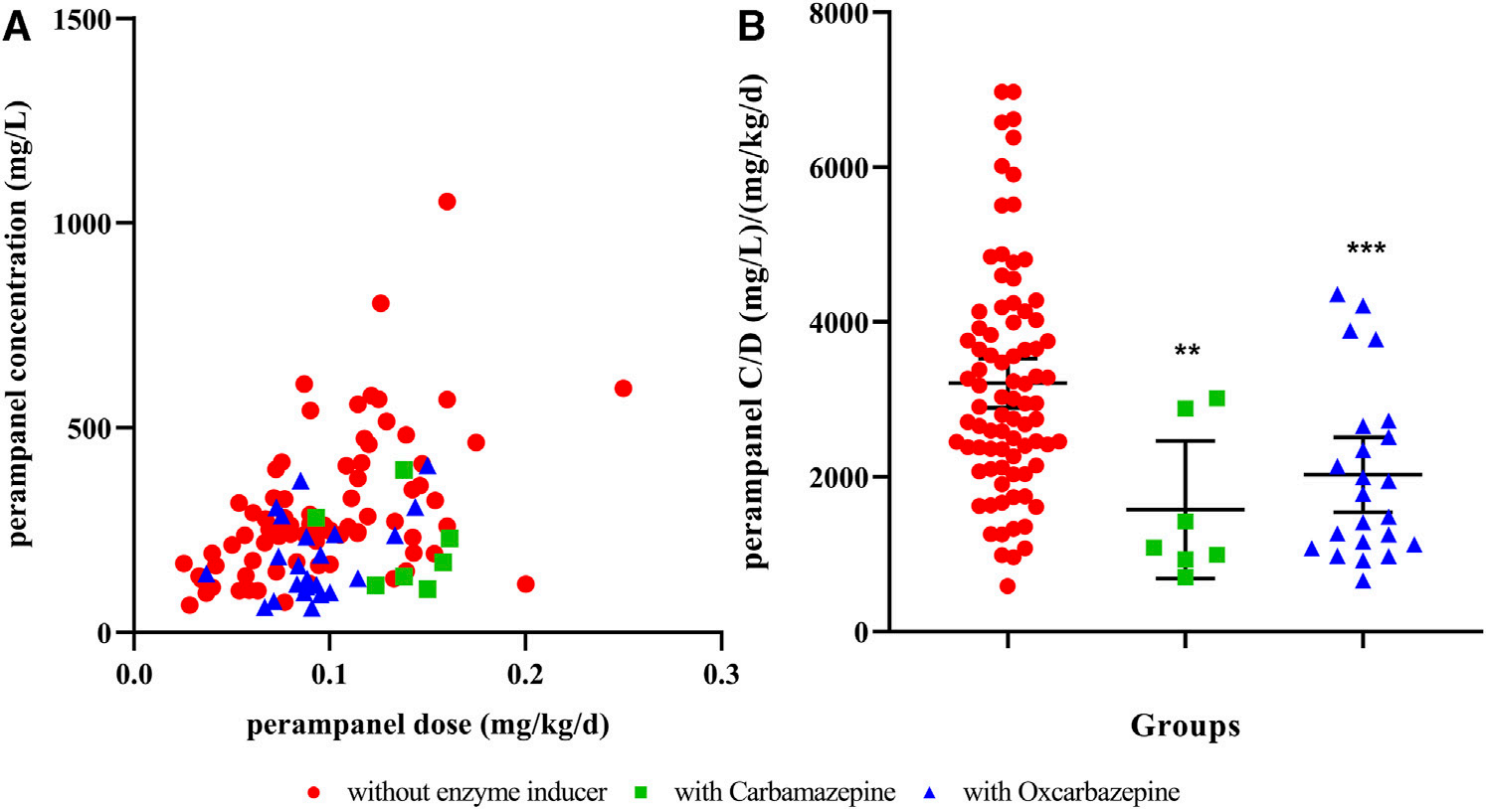
Influence of concomitant enzyme inducers on the perampanel concentration-to-dose ratio **(A)** relationship to perampanel concentration and dose; **(B)** perampanel concentration distribution in different groups. ** indicates *p* <0.01, *** indicates *p* <0.001, compared to those without enzyme inducers. C/D, concentration-to-dose ratio.

As a result, 137 pediatric patients with epilepsy were included in this study. The aged of patients ranged from 0.6 to 16.4 years and the detailed demographics are shown in Table 1. Although the concentration of PER varied greatly from 60 to 1,560 mg/L among patients, the overall therapeutic target attainment was as high as 89.8%. As shown in Table 1, the combined use of enzyme-inductive AEDs (carbamazepine and oxcarbazepine) and BMI were factors that were included in the multivariate linear regression model, thus indicating that these factors have a significant influence on the C/D of PER. When we divided the patients by concomitant AEDs, it can be seen that patients receiving carbamazepine or oxcarbazepine had significantly lower levels than other patients (Figure 1). However, from the perspective of target nonattainment, the combined use of enzyme-inductive AEDs was the only independent risk factor associated with target nonattainment (Table 2).

**TABLE 1:** Demographic characteristics of the included patients.

Variable	Total(*n* = 137)	Simple linear regression	Multivariate linear regression	
		*p*-value	Unstandardized β (95%CI)	*p*-value
Sex, n (%)				
Male	84(61.3%)	0.969		
Female	53(38.7%)			
Age (years)	8.12 ± 3.97 (0.1–16.4)	0.219		
Weight (kg)	29.8 ± 14.9 (9.0–78.7)	<0.001		
Height (cm)	127.3 ± 25.1 (68–179)	0.055		
BMI (kg/m^2^)	17.4 ± 3.28 (10.4–29.5)	<0.001	167.4(92.3, 242.6)	<0.001
Type of epilepsy				
Generalized	64(46.7%)			
Focal	68(49.6%)	0.020		0.051
Focal with generalized	5(3.65%)	0.131		0.274
Concomitant antiepileptic drugs, n (%)				
Inducers	53(38.7%)	<0.001		
Carbamazepine	7(5.11%)	0.018	−2002.5(-3101.5, −903.4)	<0.001
Oxcarbazepine	23(16.8%)	0.003	−1,053.4(-1,682.8, −424.1)	0.001
Phenobarbital	1(0.730%)	0.167		0.109
Topiramate	24(17.5%)	0.961		
Inhibitor	46(33.6%)	0.567		
Sodium Valproate	46(33.6%)	0.567		
Other	59(43.1%)	0.024		0.700
Daily dose (mg/d)	2.79 ± 1.24	0.328		
Daily dose/weight (mg/kg/day)	0.105 ± 0.046	0.016		0.448
Concentration, n (%)	285.4 ± 202.4			
<100 mg/L	12(8.76%)			
100–1,000 mg/L	123(89.8%)			
>1,000 mg/L	2(1.46%)			
C/D	2862.1 ± 1,490.0			
Laboratory data				
WBC (10^9^/L)	6.79 ± 2.11 (2.8–16.1)	0.174		0.832
HGB (g/L)	130.9 ± 11.9 (91–165)	0.258		
ALB (g/L)	43.3 ± 7.38 (34.7–108)	0.721		
ALT (U/L)	17.6 ± 9.22 (6–67)	0.213		
AST (U/L)	29.5 ± 10.3 (10–70)	0.199		0.193
TBIL (μmol/L)	7.12 ± 2.69 (2.1–19.7)	0.108		0.959

Note: Data are presented as number (percentage) or mean ± standard deviation (range).95%CI, 95% confidence interval; BMI, body mass index; C/D, concentration-to-dose ratio; WBC, white blood cell; HGB, hemoglobin; ALB, serum albumin; ALT, alanine aminotransferase; AST, aspartate aminotransferase; TBIL, total bilirubin.

**TABLE 2:** Patient demographic characteristics of patients with or without concentrations of 100–1,000 mg/L.

Variable	conc = 100-1,000 (*n* = 123)	conc<100 or conc>1,000 (*n* = 14)	Univariable logistic regression	Multivariable logistic regression	
			*p*-value	OR (95%CI)	*p*-value
Sex, n (%)					
Male	73(86.9%)	11(13.1%)	0.174		0.202
Female	50(94.3%)	3(5.66%)			
Age (years)	8.06 ± 3.87	8.64 ± 4.84	0.604		
Weight (kg)	29.6 ± 14.6	31.7 ± 18.1	0.622		
Height (cm)	127.1 ± 24.5	129.4 ± 31.4	0.748		
BMI (kg/m^2^)	17.3 ± 3.22	17.7 ± 3.93	0.694		
Type of epilepsy					
Generalized	55(85.9%)	9(14.1%)			
Focal	63(92.6%)	5(7.35%)	0.218		
Focal with generalized	5(100%)	0(0.00%)	0.999		
Concomitant drugs, n (%)					
Inducers	43(81.1%)	10(18.9%)	0.013	5.92(1.68–20.9)	0.006
Carbamazepine	7(100.0%)	0(0.00%)	0.999		
Oxcarbazepine	17(73.9%)	6(26.1%)	0.010		
Phenobarbital	0(0.00%)	1(100.0%)	>0.999		
Topiramate	21(87.5%)	3(12.5%)	0.685		
Inhibitor	43(93.5%)	3(6.52%)	0.317		
Sodium Valproate	43(93.5%)	3(6.52%)	0.317		
Other	56(94.9%)	3(5.08%)	0.098		0.961
Daily dose (mg/d)	2.88 ± 1.25	2.00 ± 0.87	0.359		
Daily dose/weight (mg/kg/day)	0.106 ± 0.044	0.088 ± 0.061	0.164		0.069
C/D	2952.7 ± 1,419.7	2066.5 ± 1884.7			
Laboratory data					
WBC (10^9^/L)	6.80 ± 2.16	6.64 ± 1.60	0.790		
HGB (g/L)	130.7 ± 11.6	132.8 ± 15.0	0.554		
ALB (g/L)	43.4 ± 7.75	42.3 ± 1.55	0.609		
ALT (U/L)	17.5 ± 9.26	18.4 ± 9.21	0.721		
AST (U/L)	29.8 ± 10.5	26.7 ± 8.8	0.317		
TBIL (μmol/L)	7.13 ± 2.48	7.06 ± 4.31	0.930		

Note: conc, concentration; OR, odds ratio; 95%CI, 95% confidence interval; BMI, body mass index; C/D, concentration-to-dose ratio; WBC, white blood cell; HGB, hemoglobin; ALB, serum albumin; ALT, alanine aminotransferase; AST, aspartate aminotransferase; TBIL, total bilirubin.

## Discussion

This was the first study to evaluate the initial therapeutic target attainment of PER in pediatric patients and risk factors associated with target nonattainment. In this study, we found that the initial target attainment of PER in pediatric patients was high (89.8%), and the couse of enzyme-inductive AEDs was an independent risk factor for PER target nonattainment. These findings would benefit the clinical application of PER in pediatric patients.

The variance in plasma PER concentrations was large in our study, which was also elucidated by other studies. Steinhoff et al. found a large concentration range of 19–2436 ng/mL in adults (Steinhoff et al., 2019). However, the target attainment of PER in our study was as high as 89.8%. Li et al. also found a high therapeutic target attainment in pediatric patients (75%) using a narrower reference range (180–610 mg/L) (Li et al., 2022). Several reference ranges for PER have been reported. A previous pharmacokinetic-pharmacodynamic study suggested a PER concentration of 70 ng/mL or greater for efficacy in adults and adolescents, which was lower than our lower boundary (Gidal et al., 2013). However, PER concentrations have ranged from 180 to 980 μg/L in the patients who responded to PER in these trials, and this range was used as a putative reference range (Patsalos et al., 2018). Ranges of 50–400 and 200-600 were also suggested, but without sufficient support (Johannessen Landmark et al., 2020; Yamamoto et al., 2020). The Norwegian Association of Clinical Pharmacology established national guidelines about AED TDM for Norway and suggested a reference range of 100–1,000 ng/mL for PER based on current available evidence (Reimers et al., 2018). Previous study also confirmed good tolerability in pediatric patients in middle and long-term therapy of PER (Operto et al., 2020). Thus, the reference range used in the current study was reasonable. From the results of the study, routine TDM in pediatric patients to ensure that the PER concentration is in the reference range may be unnecessary. However, using a TDM method to identify individual optimized PER levels or investigate patient adherence to PER is still meaningful.

PER is extensively metabolized by CYP3A, and naturally, its pharmacokinetics are affected by enzyme inducers or inhibitors (Rogawski and Hanada, 2013). Concomitant use of carbamazepine and oxcarbazepine was negatively correlated to PER C/D in our study. This is in accordance with previous reports that the concomitant use of enzyme-inducing AEDs could result in lower PER concentrations or C/D, both in children and adults (Patsalos et al., 2016; Gaudio et al., 2019; Ikemoto et al., 2019; Ishikawa et al., 2019). Carbamazepine could increase the clearance of PER in a population pharmacokinetic analysis (Fujita et al., 2022). However, the influence of the enzyme inhibitor AED, valproic acid, was not identified in this study. Valproic acid was reported to affect PER C/D in adults and adolescents. However, a previous study also showed that the effect of coadministration of the CYP3A inhibitor ketoconazole on the pharmacokinetics of PER could be neglected (Gidal et al., 2017). Our study found that only one personnel characteristic, BMI, was related to C/D. While age was also reported to be a factor of C/D (younger and older than 12 years) (Ikemoto et al., 2019), Patsalos et al. also found that age was independent of PER plasma concentration in adults (Patsalos et al., 2016). Although numerous factors can affect PER C/D, it is uncertain whether this influence had clinical significance until this report.

For now, there is no ethnic-related differences founded in the pharmacokinetics of PER (de Biase et al., 2019). A population pharmacokinetic analysis including phase Ⅱ/Ⅲ trails data found that the difference of clearance between Asian race and other the was small and clinical irrelevant (Takenaka et al., 2018). Another pharmacokinetic study also concluded that there were no clinically relevant ethnic differences in PK following multiple doses of perampanel between Korean, white, or Japanese subjects (Tabuchi et al., 2018).

The advantage of our study is that concomitant use of AEDs was found to be the only risk factor associated with PER therapeutic target nonattainment in pediatric patients. This would be helpful in clinical practice. Physicians need to be aware that when prescribing PER, the dose should be elevated or adjusted according to TDM results if concomitant enzyme-inductive AEDs are present. It should be noted that in the multivariate logistic regression, concomitant use of inducers and use of oxcarbazepine were related, and we selected the former to perform the final analysis. It is reasonable that carbamazepine was reported to have a larger inductive effect on PER metabolism than oxcarbazepine (Patsalos, 2015).

This study also has some limitations. The international consensus about the therapeutic range of PER has not yet been reached; thus, the target attainment of PER would change when the reference range changes. PER is highly bounded to plasma proteins but free PER concentrations were not determined in this study. Clinical outcome, as well as the relationship between PER concentration and outcome, were not evaluated. Patients receiving some enzyme-inductive AEDs, especially weak enzyme-inductive AEDs such as topiramate, are few, and the influence of these drugs on PER target attainment should be evaluated in future studies.

## Conclusion

In pediatric patients with epilepsy, PER concentrations varied to a great extent, but the target attainment was high if using the reference range of 100–1,000 ng/mL. BMI and the couse of enzyme-inductive AEDs significantly affected the C/D of PER. However, only the concomitant use of enzyme-inductive AEDs was associated with PER therapeutic target nonattainment. Routine TDM for these patients to ensure PER concentration may be unnecessary, except for those receiving combined enzyme inductive AEDs.

## Data Availability

The original contributions presented in the study are included in the article/Supplementary material, further inquiries can be directed to the corresponding authors.
